# In vivo TSPO and cannabinoid receptor type 2 availability early in post-stroke neuroinflammation in rats: a positron emission tomography study

**DOI:** 10.1186/s12974-017-0851-4

**Published:** 2017-03-29

**Authors:** Teruyo Hosoya, Dai Fukumoto, Takeharu Kakiuchi, Shingo Nishiyama, Shigeyuki Yamamoto, Hiroyuki Ohba, Hideo Tsukada, Takatoshi Ueki, Kohji Sato, Yasuomi Ouchi

**Affiliations:** 1 Department of Neuroanatomy and Neuroscience, Hamamatsu University School of Medicine, 1-20-1 Handayama, Higashi-ku, Hamamatsu, 431-3192 Japan; 2 Central Research Laboratory, Hamamatsu Photonics KK, 5000 Hirakuchi, Hamakita-ku, Hamamatsu, 434-8601 Japan; 3 Department of Biofunctional Imaging, Preeminent Medical Photonics Education & Research Center, Hamamatsu University School of Medicine, 1-20-1 Handayama, Higashi-ku, Hamamatsu, 431-3192 Japan

**Keywords:** Microglia, Cannabinoid receptor type 2, Translocater protein, Neuroinflammation, Positron emission tomography, Immunostaining, Early stroke

## Abstract

**Background:**

Upregulated levels of 18-kDa translocator proteins (TSPO) and type 2 endocannabinoid receptors (CB2) are considered to reflect different aspects of microglia-related neuroinflammatory responses in the brain. Relative to the increase in the TSPO expression that occurs slightly later during neuroinflammation in a proinflammatory fashion, CB2 activation is considered to relate to the neuroprotective responses that occurs predominantly at an early stage of brain disorders. These findings, however, were deduced from studies with different animal samples under different experimental settings. Here, we aimed to examined the differences in TSPO binding and CB2 availability at an early stage of stroke in the same animal using positron emission tomography (PET).

**Methods:**

We used a total of eight Sprague-Dawley rats that underwent photothrombotic stroke surgery. The binding levels of a TSPO tracer [^11^C](*R*)PK11195 and a CB2 tracer [^11^C]NE40 were measured at 24 h after the surgery in the same animal using PET in combination with immunohistochemistry for CB2 and several other markers. A morphological inspection was also performed with X-ray computed tomography for small animals.

**Results:**

The levels of [^11^C]NE40 binding potential (BP_ND_) were significantly higher in the cerebral cortical region on the lesion side than those on the non-lesion side, whereas no difference was found in the levels of [^11^C](*R*)PK11195 BP_ND_ between hemispheres. The tracer influx index (R1) data were all reduced on the lesion side irrespective of tracers. This increase in [^11^C]NE40 BP_ND_ was concomitant with an elevation in CB2 expression mainly within the microglia in the peri-infarct area, as shown by immunohistochemical examinations with Iba-1, CD11b/c+, and NG2+ staining.

**Conclusions:**

The present results provide in vivo evidence of different responses of microglia occurring in the acute state of stroke. The use of the CB2 tracer [^11^C]NE40 allows us to evaluate the roles played by the neuroprotective aspect of microglia in acute neuroinflammatory processes.

**Electronic supplementary material:**

The online version of this article (doi:10.1186/s12974-017-0851-4) contains supplementary material, which is available to authorized users.

## Background

Microglia change morphologically and functionally from their resting state to their activated state in response to several neuroinflammatory and neurodegenerative diseases. In other words, microglial activation accompanies any damage to the brain environment. This is one reason why activated microglia represent an important marker of neuroinflammation [1]. Specifically, non-invasive imaging of activated microglia is a useful tool to detect in vivo neuroinflammatory disease. To date, the first-generation translocator protein 18 kDa (TSPO) marker [^11^C](*R*)PK11195 has been widely used as a PET radioligand for that purpose. PET studies using [^11^C](*R*)PK11195 have been carried out on patients with several neuroinflammatory diseases such as Alzheimer’s disease (AD) [2], Parkinson’s disease (PD) [3], and Huntington’s disease [4]. In addition, ischemic stroke causes direct insults to brain tissue and the immediate activation of microglia, which can also be visualized on PET with [^11^C](*R*)PK11195 [5]. This is an experimental advantage in studies on time course changes in microglial activation, unlike animal models for chronically developed neurodegenerative brain disorders. Specifically, since stroke is a sudden onset disease, an early depiction of the extent at which the brain is compromised is important for treatment to delay the disease progression. In this context, TSPO imaging may be preferable. To date, it is well documented that the accumulation of [^11^C](*R*)PK11195 or the second-generation tracer [^18^F]DPA714 is reported to occur relatively later after brain injury resulting from toxin injection [6] or traumatic insults [7, 8].

The imaging of endocannabinoid receptor type 2 (CB2) has recently been considered to be an alternative method for targeting activated microglia using PET imaging. The endocannabinoid system in the central nervous system has been identified to provide neuroprotective effects following brain injury. In particular, CB2 is upregulated in microglia during neurodegenerative and neuroinflammatory diseases such as AD [9–11], multiple sclerosis [12], PD [13] and ischemia [14–17], and is associated with microglial activity [18]. Moreover, the administration of a selective CB2 agonist reduced infarct volume and improved motor function scores [16]. Recently, a PET tracer that binds specifically to CB2 was developed to illustrate CB2 availability in vivo [19]. Although it was reported that the binding of [^11^C]NE40 24 h after ischemic injury using a photochemically induced thrombosis (PIT) technique failed to increase [20], six out of nine PET data were not corrected for the attenuation of radioactivity, and the correction is critical for the quantification of PET data. Considering the early-developed neuroprotective role of microglia, a failure to detect the elevation of [^11^C]NE40 uptake in AD patients [21] is understandable because the activated microglia during the chronic state of neurodegeneration are considered to be acting as proinflammatory agents [22]. Therefore, the stroke model might be suitable for evaluating the early response of microglia because the insult can be controlled purposefully.

In neuroinflammation, microglia are considered to play a dual role in their function. Classically activated M1 phenotype microglia release proinflammatory mediators, whereas alternatively activated M2 phenotype microglia enhance phagocytic activity and reduce the production of inflammatory mediators [23, 24]. In our previous study, we demonstrated that neuronal damage occurred on days 1, 3, 7, and 14 after PIT using [^11^C](*R*)PK11195 [25]. The uptake peak of [^11^C](*R*)PK11195 was found on day 7 after PIT treatment and overlapped with the high immunoreactivity area of microglial marker Iba1, suggesting that the uptake of [^11^C](*R*)PK11195 reflects microglial activity. However, it is difficult to distinguish between neurotoxic M1 and neuroprotective M2 microglia phenotypes on PET imaging with [^11^C](*R*)PK11195. Then, we focused on [^11^C]NE40, which targets CB2, with the goal of observing neuroprotective microglia at an acute stage of brain injury.

Therefore, the purpose of the present study was to focus on the early phase of stroke and to elucidate the different binding patterns of CB2 and TSPO tracers in a very acute stage of brain insult, such as stroke, by comparing the levels of [^11^C]NE40 binding with those of [^11^C](*R*)PK11195 binding in rats after PIT surgery. These in vivo data were evaluated with immunohistochemistry for CB2, TSPO, and several cell type-specific markers after completion of the PET measurements, resulting in cessation of the in vivo study with the same animals.

## Methods

### Animals

Eight 8-week-old male Sprague-Dawley rats (250–300 g) purchased from the SLC Company (Hamamatsu, Japan) were used in this study. They were housed in cages with free access to food and water. All animal protocols and the following experiments were approved by the Ethics Committees of the Central Research Laboratory at Hamamatsu Photonics and Hamamatsu University School of Medicine. In addition, all applicable institutional and/or national guidelines for the care and use of animals were followed.

The PIT procedure was performed as reported previously [26]. Briefly, the rats were anesthetized and maintained with 2% halothane in a mixture of 70% room air and 30% O_2_ throughout the following procedure. After the insertion of an infusion line into the tail vein, the scalp and temporal muscle were flipped, and then a subtemporal craniotomy was performed. The main trunk of the left middle cerebral artery (MCA) was observed through the dura mater under an operating microscope through a window that was anterior to the foramen of the mandibular nerve. After the intravenous infusion of 20 mg/kg of rose bengal (Wako Pure Chemical Industry, Osaka, Japan), photoillumination was performed utilizing a green light at 540 nm (model L4887, Hamamatsu Photonics, Hamamatsu, Japan), which was delivered to the MCA through the dura mater for 10 min using a 3-mm optic fiber placed onto the window within the skull base. After confirming thrombotic occlusion of the MCA, the incision was closed. The animals were then allowed to awake from anesthesia and were returned to their cages.

### PET measurements

We utilized a high-resolution animal PET scanner (SHR-38000, Hamamatsu Photonics, Japan) under an axial field of view (FOV) of 330 mm, a transaxial FOV of 108 mm, and a transaxial spatial resolution of 2.3 mm in the center. Eight animals were scanned twice a day using PET with [^11^C](*R*)PK11195 and [^11^C]NE40 beginning 24 h after PIT. The interval between the two scans was set to 2 h, and the order of the scans was counterbalanced. The animals were anesthetized using an initial dose of chloral hydrate (400 mg/kg, i.p.) followed by a continuous infusion of chloral hydrate (100 mg/kg/h, i.v.) during the entire imaging experiment. They were placed in the prone position on a fixation plate and then set within the gantry hole of the PET scanner. After a 15-min transmission scan utilizing an external ^68^Ge/^68^Ga rod source (67 MBq) for attenuation correction, a serial emission scan that lasted for 60 min was performed immediately following each tracer injection of [^11^C](*R*)PK11195 or [^11^C]NE40 at a dose of 48 MBq/kg; tracers were injected intravenously through the cannula that was inserted into the tail vein. The specific activity of each tracer used was above 50 GBq/μmol. No arterial sampling was conducted. The PET data were reconstructed using 3D DRAMA (iteration 2, gamma 0.1) with a Gauss filter of 1.0 mm in full width at half maximum (FWHM), yielding a voxel size of 0.65 × 0.65 × 1.0167 mm for the reconstructed image. To obtain the anatomical information, X-CT scans were performed immediately following PET measurement using a ClairvivoCT (Shimadzu Corporation, Kyoto, Japan).

### Data analysis and statistics

Using an image analysis software (PMOD, version 3.1; PMOD Technologies Ltd, Zurich, Switzerland), we estimated the levels of BP_ND_ (an availability of TSPO and CB2) and R1 (a tracer influx index according to blood flow: a ratio of K1/K′1, or an influx in target of interest/an influx in reference of interest) for [^11^C](*R*)PK11195 and [^11^C]NE40 based on a simplified reference tissue model [27, 28] and then created the parametric brain images of BP_ND_ and R1 (a tracer uptake index). During this process, the time-activity curve from the bilateral cerebellar cortex was used as a reference input function because the infratentorial brain region might be less affected by the MCA occlusion event; however, functional connection emerging as diaschisis could be a possibility of the confounding factor. The selection of the intact contralateral cortical region as a reference region as conducted elsewhere [20] seems inadequate because the numbers of the CD11b+/CD3+ cells (for microglia) were comparable in ischemia-affected and non-affected hemispheres 24 h after stroke [29].

As described elsewhere [30, 31], the elliptical regions of interest (ROIs), ranging from 12 to 24 mm^2^ wide, were symmetrically placed in the bilateral brain regions covering the peri-infarct area mainly in the frontal and parietal cortices by referring to the X-CT images, on which the coronal slices of the frontal and parietal cortices were determined according to the Paxinos rat brain stereotactic atlas [32] (see Additional file 1: Figure S1).

Student’s *t* test statistics were used to compare the conditions and the significance level was set at *p* < 0.05 with a correction for multiple comparisons because multiple loci were chosen.

### Immunohistochemistry

The rats were anesthetized with chloral hydrate (400 mg/kg) and then transcardially perfused with saline followed by 4% paraformaldehyde (PFA) (pH 7.4). The brains were removed, post-fixed in 4% PFA, and immersed in cryoprotectant solution (30% sucrose in 0.1 M phosphate buffer) until the tissue sank. Tissues were frozen in dry ice and stored at −80 °C until they were used. Twenty-micrometer frozen coronal sections were cut using a cryostat. The slides were blocked with 5% goat serum in PBS containing 0.1% Triton X-100 for 1 h at room temperature (RT) and then incubated with primary antibodies for 2 h at RT. After being washed, the slides were then incubated for 2 h at RT with secondary antibodies. The following primary antibodies were used in this study: mouse anti-GFAP (1:500, Millipore), rabbit anti-Iba1 (1:1000, Wako), mouse anti-CD11b/c (1:500, DB Pharmingen), mouse anti-NG2 (1:200, Millipore), rabbit anti-cannabinoid receptor 2 (1:100, Abcam), and rabbit anti-rat TSPO (1:200, Biobyt). The following secondary antibodies were used in this study: Alexa Fluor 488 anti-rabbit-IgG and Alexa Fluor 594 anti-mouse-IgG (1:500, Invitrogen).

## Results

### PET findings

**Fig. 1:**
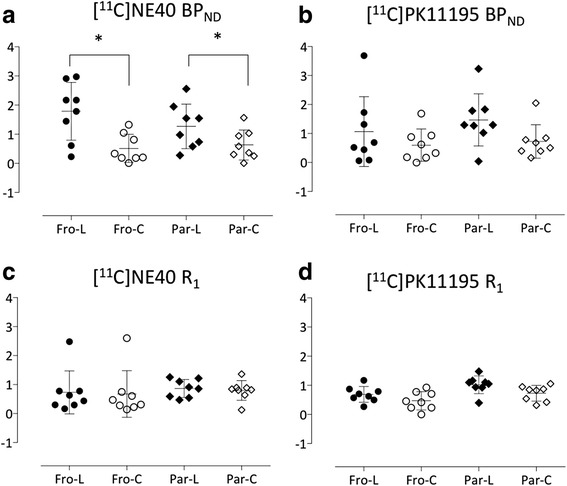
The levels of BP_ND_ (**a**, **b**) and R1 (**c**, **d**) in each brain region in [^11^C]NE40 and [^11^C](R)PK11195 studies, respectively. Only BP_ND_ of [^11^C]NE40 was significantly higher in the affected cortical regions compared to the intact regions (*p < 0.05)

The Student’s *t* test showed a significantly higher level of [^11^C]NE40 BP_ND_ in the affected regions of the frontal cortex (*t* = 3.54, *p* < 0.05 corrected for multiple comparisons) and parietal cortex on the lesion side (*t* = 3.21, *p* < 0.05 corrected) than [^11^C](*R*)PK11195 BP_ND_ (Table 1, Fig. 1a, b). In contrast, the R1 value that reflects the degree of tracer influx depending on regional blood flow was frequently observed to be low in the MCA territory, regardless of the tracer used (Fig. 1c, d), suggesting that the influxes of these two different tracers might be equally damaged in this experimental setting. An additional examination using tracer-summation PET images (standardized uptake value, SUV) failed to show this significance although the similar trend was found (Additional file 2: Figure S2).

**Table 1:** Differences in the levels of BP_ND_ and RI values

	Region	[^11^C]NE40		[^11^C](*R*)PK11195	
		Lesion	Contralateral	Lesion	Contralateral
BP_ND_	Frontal	1.79 ± 0.99*	0.51 ± 0.48	1.07 ± 1.20	1.47 ± 0.90
	Parietal	1.27 ± 0.77*	0.63 ± 0.51	0.60 ± 0.56	0.73 ± 0.58
R1	Frontal	0.73 ± 0.74	0.87 ± 0.31	0.68 ± 0.80	0.80 ± 0.34
	Parietal	0.68 ± 0.27	1.02 ± 0.30	0.47 ± 0.31	0.73 ± 0.27

*p < 0.05 vs contralateral (non-lesion) side

**Fig. 2:**
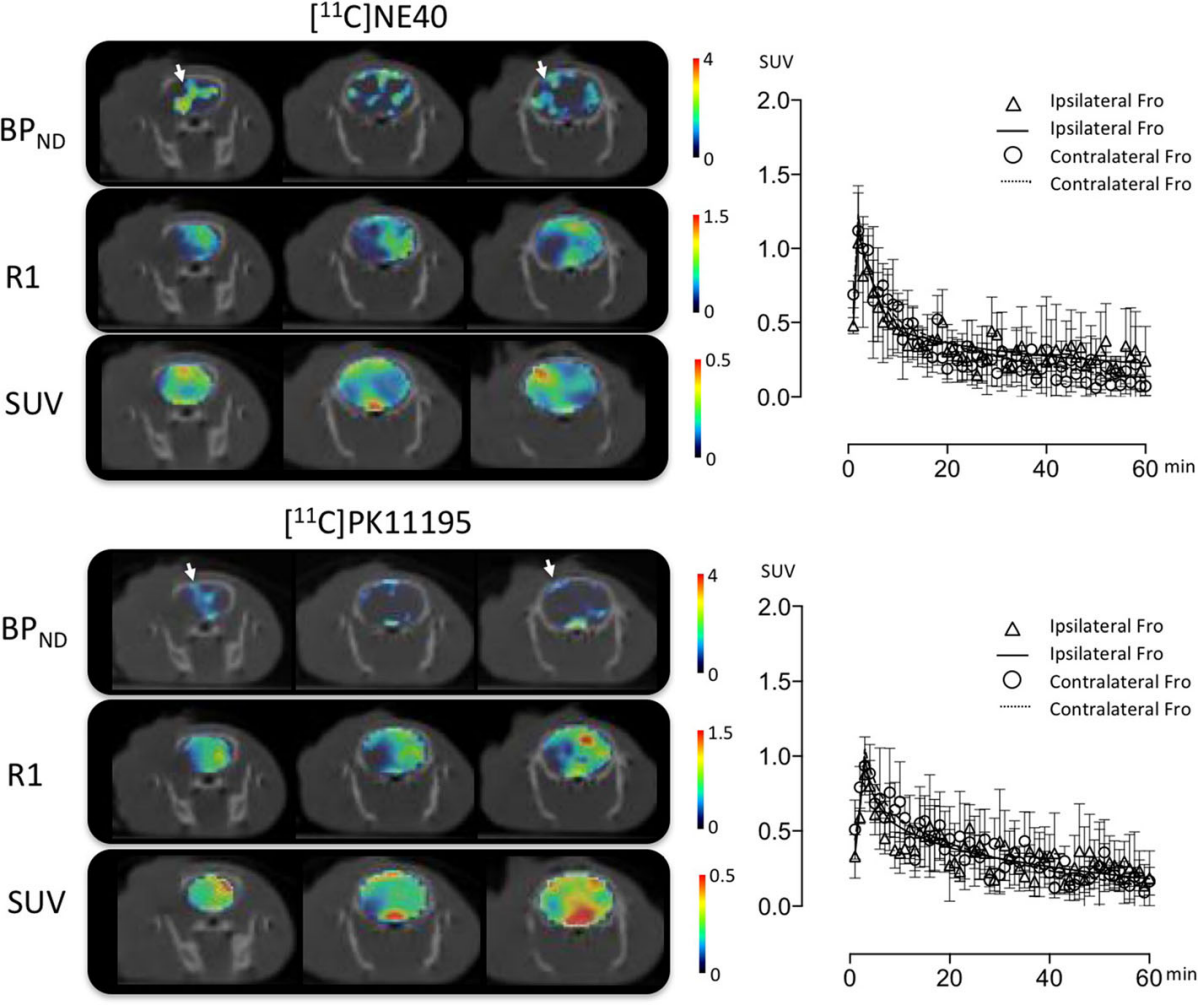
Coronal parametric images of BP_ND_, R1, and SUV (standardized uptake value) superimposed on X-CT images in [^11^C]NE40 and [^11^C](R)PK11195 studies. A SUV image was generated as a summation of tracer uptake during 40–60 min post-injection. The *arrows* indicate the peri-infarct region (penumbra). Mean tissue time-activity curves with standard deviations of each tracer on both frontal cortices are shown in different panels. Ipsilateral, lesion side; contralateral, non-lesion side

As shown in Fig. 2, higher uptake of [^11^C]NE40 was observed in the peri-infarct region (penumbra, arrow) than the [^11^C](*R*)PK11195. Although the difference in time course of the tracer uptake (the tissue time-activity curve, TAC) was small between [^11^C]NE40 and [^11^C](*R*)PK11195, there was a gradual increase in [^11^C]NE40 TAC on the ipsilateral (infarct) side later during the scan, while no difference was observed in [^11^C](*R*)PK11195 TACs on either side.

### Immunohistochemical findings

**Fig. 3:**
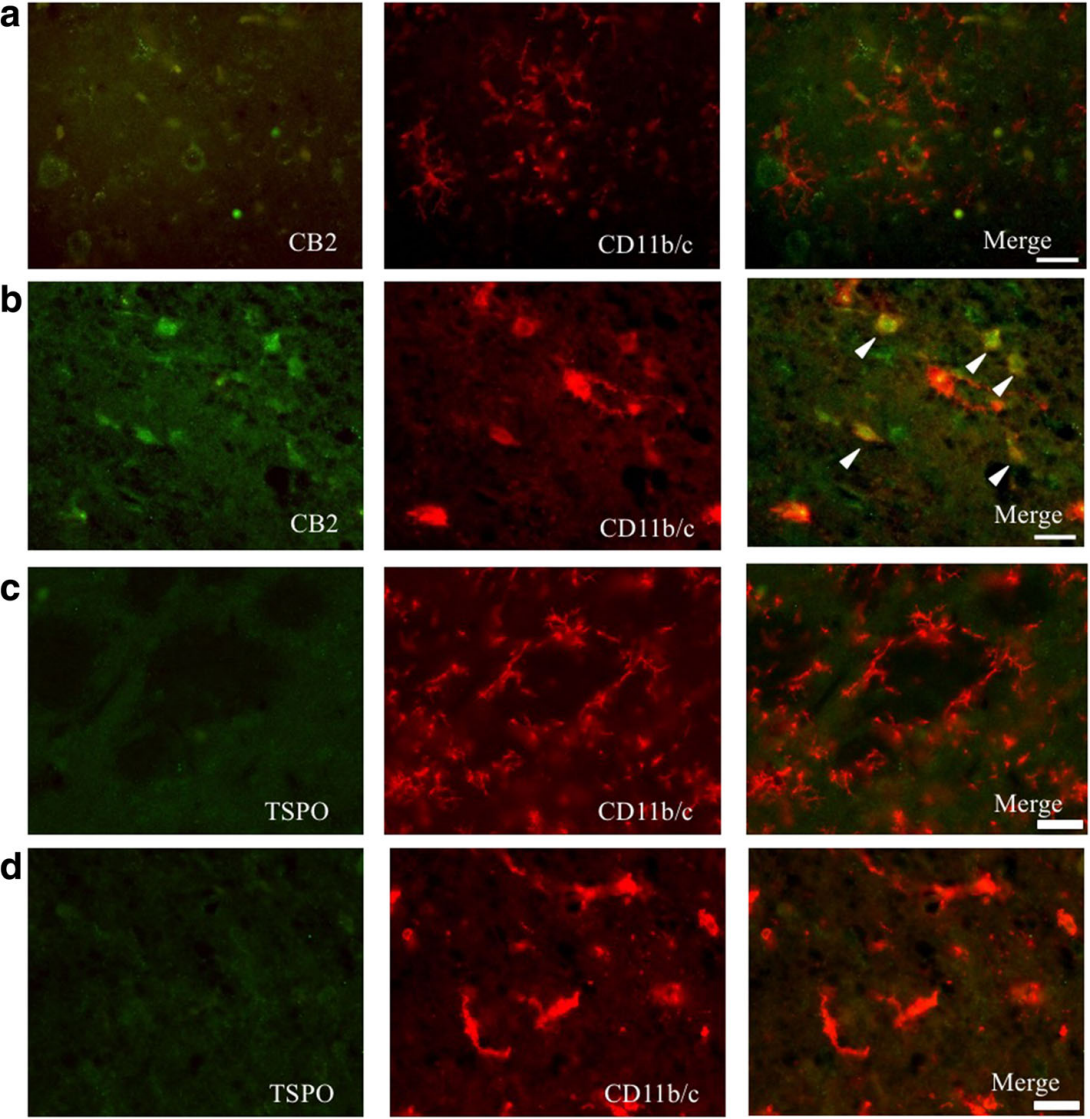
Double-immunostaining for CB2 and CD11b/c in the contralateral (non-lesion) (**a**) and the peri-infarct (**b**) areas and for TSPO in the contralateral (**c**) and peri-infarct (**d**) areas on day 1 after PIT treatment. Several microglia were co-stained for CB2 and CB11b/c (*arrowhead*). *Scale bar*, 25 μm

The brain section images revealed that large ischemic lesions occurred from the striatum to the cortical region in our experimental model. Within the infarct core, only cell debris was observed (Additional file 3: Figure S3). As shown in Additional file 3: Figure S3 and Fig. 3, Iba-1-positive cell and CD11b/c+ cell regions were predominantly found within the peri-infarct region, while GFAP-positive astrocytes were found to be scattered throughout the brain.

**Fig. 4:**
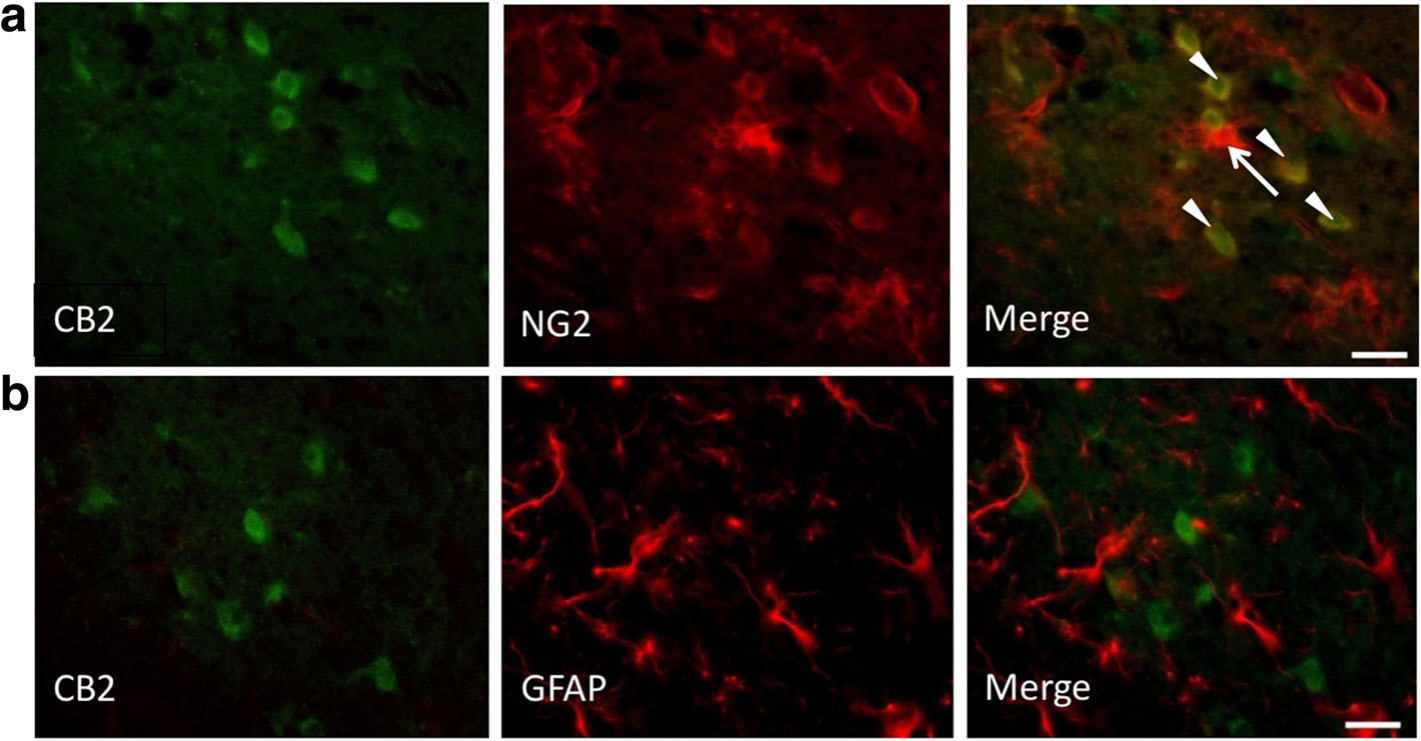
Double-immunostaining for CB2 (*green*) and GFAP (*red*) in the peri-infarct area (**a**). NG2+/CB2+ cells were round (*arrowhead*) and NG2+/CB2− cells were in a ramified form (*arrow*). **b** Double-immunostaining for CB2 (*green*) and NG2 (*red*) in the peri-infarct area on day 1 post PIT treatment. *Scale bar*, 25 μm

Co-staining for CB2 and the microglial marker CD11b/c showed that CD11b/c+ microglia in the contralateral (non-lesion) area were in their resting state with ramified processes, while they were negative for CB2 on day 1 after PIT treatment (Fig. 3). It appeared that microglial processes surrounded the CB2+ cells. In the peri-infarct area, some CD11b/c+ cells were co-labeled with CB2; their form exhibited a round shape (Fig. 3, arrowhead). Co-staining for TSPO and CD11b/c shows that microglia did not express TSPO (Fig. 3). TSPO was negative in all areas. Because no GFAP-positive astrocytes were co-labeled with CB2, GFAP+ cells were unlikely to be associated with CB2+ cells (Fig. 4).

Because NG2 (neural/glial antigen 2) was reported to be highly expressed at an early state after brain injury [33] and in the activated microglia and infiltrated macrophages in brain insults [34–37], we also performed co-staining for CB2 and NG2 to determine whether CB2 in the peri-infarct area was expressed by activated NG2+ cells. The NG2 signal was very strong in regions adjacent to the infarct area and some NG2-positive cells were co-labeled with CB2 (Fig. 4). Notably, CB2+/NG2+ cells were round in shape (Fig. 4, arrow head) and CB2−/NG2+ cells exhibited a ramified form with thin processes (Fig. 4, arrow).

**Fig. 5:**
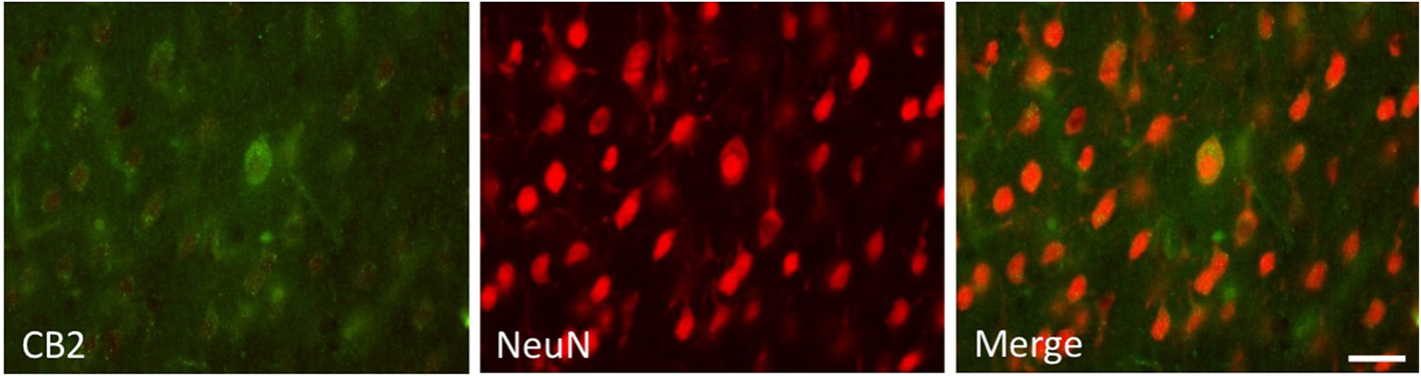
Double-immunostaining for CB2 and NeuN in the contralateral cortex. Some NeuN+ cells were weakly co-labeled with CB2. *Scale bar*, 25 μm

Co-staining with CB2 and the neural marker NeuN showed that some NeuN+ cells in the contra- and ipsilateral cortices were weakly co-labeled with CB2 (Fig. 5). However, in the peri-infarct area, no NeuN+ cells were co-labeled with CB2. Therefore, the uptake of [^11^C]NE40 in the contralateral cortex might reflect some degree of its binding to CB2 on neurons.

## Discussion

### Microglial activation detection using the CB2 tracer

We found an increase in [^11^C]NE40 binding and elevated CB2 availability specifically in the peri-infarct area at an early stage after ischemic injury induced by the PIT method. This finding was consistent with our immunohistochemical results that showed co-localization of CD11b/c+ and CB2-positive cells exhibiting microglia with a round shape, suggesting that some CD11b/c+ cells expressed CB2 in regions adjacent to the infarct area. This immunohistochemical finding is also in line with the previous observation that a number of CD11b+/CD3+ cells (for microglia) were found in ischemia-affected areas 24 h after stroke [29]. A previous report suggested that CB2 can be detected in the “responsive” and “primed” states but not in the “resting” state of microglia in vitro [38]. Thus, microglia on day 1 after PIT treatment might be at an early stage in the inflammatory process, being in either the “responsive” or “primed” state in vivo. In contrast, the binding of [^11^C](*R*)PK11195 was not co-localized in areas with high [^11^C]NE40 binding. Because TSPO tracers such as [^11^C](*R*)PK11195 are considered to be PET probes of delayed microglial activation as shown in literature [22], the presence of [^11^C]NE40 uptake along with a lack of [^11^C](*R*)PK11195 uptake suggests that CB2 may be more implicated than TSPO during the early-phase activation of microglia in the post-stroke brain region.

### Differences in binding of TSPO and CB2 tracers

In our previous study, we utilized [^11^C](*R*)PK11195 and studied its uptake at several time points in the PIT model; furthermore, by utilizing immunostaining for Iba1, high [^11^C](*R*)PK11195-uptake areas appeared to correspond to high microglial activity areas on day 7 after PIT treatment [25]. In this study, we demonstrated that [^11^C]NE40 is a suitable target for a PET tracer detecting neuroinflammation developed soon after brain ischemic insult because the uptake of [^11^C]NE40 is higher than that of [^11^C](*R*)PK11195 1 day after PIT treatment. To date, [^11^C](*R*)PK11195 has primarily been used for the imaging of inflammatory conditions such as neurodegenerative diseases. In the chronic stage of inflammation [6], neurotoxic M1 phenotype microglia are dominant and the TSPO tracer [^11^C](*R*)PK11195 reflects inflammatory rather than anti-inflammatory microglial activity [22]. To support this contention, we recently showed an elevation in the second-generation TSPO tracer [^11^C]DPA713 for illustrating chronically activated microglia under inflammatory circumstances [39].

### Role of microglia in activation

Some studies have indicated that microglial CB2 is neuroprotective both in vivo and in vitro. The administration of the CB2 agonist JWH-133 in an ischemic rat brain model reduced ischemic volume, promoted the production of anti-inflammatory mediators, and suppressed the release of inflammatory mediators and Iba1 expression in microglia [15]. In an in vitro experiment, the administration of anandamide (a kind of endocannabinoids) to lipopolysaccharide-treated microglia reduced the activation of M1 phenotype microglia and elevated anti-inflammatory factors [40]. Overexpression of CB2 causes mice to have resistance to depression after stress [41] and intraperitoneal administration of a CB2 agonist can ameliorate anxiolytic effects [42]. Thus, the present PET data that show a higher accumulation of [^11^C]NE40 and lower binding of [^11^C](*R*)PK11195 at an early stage after PIT-induced stroke indicate possibly different roles of microglia in terms of neuroprotective or neuroinflammatory content during early brain injury.

### Immunostaining and PET findings

The current immunohistological study showed that the CB2+ cells were surrounded by microglial processes and some CD11b/c+ cells with a round morphology were co-labeled with CB2 in the peri-infarct area. In addition, CB2-positive cells were weakly co-labeled with NeuN in the contralateral cortex and CB2+/NeuN+ neurons were surrounded by CB2+ cell processes. The latter finding might induce a slight upregulation of [^11^C]NE40 on the contralateral side, which might explain why no GFAP+ cells were co-labeled with CB2. The immunostaining finding that CB2-colabelled NG2+ cells were found to be round in shape suggested that they were monocytes and played a protective role [43]. In contrast, NG2+ cells without co-labeling with CB2 were thought to be oligodendrocyte precursor cells insensitive to CB2. It appears that astrocytes are not associated with CB2 1 day after PIT treatment, even though GFAP+ cells were co-labeled with CB2 on day 3 after ischemic injury [44]. Day 1 after injury may be too early for astrocytes to respond because the activation of astrocytes is considered to occur at a late stage of the inflammatory process. Although the TSPO tracer [^11^C](*R*)-PK11195 is believed to bind not only to activated microglia but also to activated astrocytes, either [^11^C]NE40 or [^11^C](*R*)PK11195 may not have captured astrocyte activation during the early stages of injury in the current experiment. Incompatible with the low level of [^11^C](*R*)-PK11195 binding, the TSPO immunohistochemical staining exhibited a negligible level of TSPO expression in microglia in the ipsilateral lesion area.

### Limitations

First, we measured changes in the uptake of both [^11^C]NE40 and [^11^C](*R*)PK11195 24 h after surgery. These data cannot elucidate the timing of the state switch, dominancy, protective activity, or inflammatory processes of microglia. Different methods for acute stroke (transient or permanent ischemia) would generate different outcomes in the ischemic region with regard to inflammatory substances and cells as shown previously [29, 44]. According to these reports, the present PIT technique, one of the permanent stroke models, would yield less damage than transient, reperfusion model. As for microglia, not number but morphology of microglia was reportedly changed 1 day after permanent stroke [44]. Despite these findings, further study is needed to address changes in time course of inflammatory events after stroke. Second, because the cerebellum was chosen as the reference region to calculate BP_ND_ in this study, vascular injury may affect each region of the brain on the theory of diaschisis. However, the cerebellar PET counts were averaged between both sides in this study to minimize these possible diaschisis effects. Therefore, the estimated BP_ND_ values could reflect tissue tracer binding at target regions. Third, because the spatial resolution of the PET scanner used was relatively large (2.3 mm), there might be an effect of partial volume on the results. To reduce this effect, we tried to set ROIs that measured areas twice as large as the FWHM of the scanner, or we may choose autoradiography instead of PET although the parameters changing in the same animal cannot be obtained in autoradiography.

## Conclusions

The present increase in [^11^C]NE40 binding, but not [^11^C](*R*)PK11195 binding concomitant with a higher CB2 immunochemical expression within the microglia over the peri-infarct region early after PIT injury, indicates that acutely activated microglia in early-stage ischemic stroke might be involved in the neuroprotective process of neuroinflammation. While a recent new CB2 tracer [^11^C]A836339 has been reported to exceed the sensitivity of [^11^C]NE40 to bind to CB2 under the chronic state of neurodegeneration in vivo [9], the present result suggests that [^11^C]NE40 might be adequate for depicting activated microglia at a very early stage of brain disorders.

## Additional files

Additional file 1: Figure S1. CT images and regions of interest. Frontal, −2 mm from the olfactory-frontal cortex junction (nearly 3 mm from the bregma). Parietal, −6 mm from the junction (nearly −1 mm from the bregma). Blue, lesion side; light blue, contralateral (non-lesion) side. (JPG 68 kb)

Additional file 2: Figure S2. The levels of SUV in each brain region. No significant difference was found in either [^11^C]NE40 or [^11^C](R)PK11195 SUV levels. (JPG 100 kb)

Additional file 3: Figure S3. Immunohistochemical results one day after photochemically induced thrombosis (PIT). Iba-1- and GFAP-positive regions are scattered around the infarct core. (JPG 248 kb)

## Abbreviations

^11^C-(*R*)PK11195: (*R*)-*N*-(11)C-Methyl-*N*-(1-methylpropyl)-1(2-chlorophenyl)isoquinoline -3-carboxamide
^11^C-DPA713: *N*,*N*-Diethyl-2-[2-(4-methoxyphenyl)-5,7-dimethylpyrazolo[1,5-a]pyrimidin-3-yl]acetamide
^11^C-NE40: 2-Oxo-7-[11C]methoxy-8butyloxy-1,2-dihydroquinoline-3-carboxylic acid cyclohexylamide
AD: Alzheimer’s disease
BPND: Binding potential non-displaceable
CB2: Type 2 endocannabinoid receptor
CD11b: An integrin family member
GFAP: Glial fibrillary acidic protein
MCA: Middle cerebral artery
NeuN: Neuronal nuclei
NG2: Neural/glial antigen 2
PD: Parkinson’s disease
PET: Positron emission tomography
PFA: Paraformaldehyde
PIT: Photochemically induced thrombosis
R1: Tracer influx index
RT: Room temperature
TAC: Time-activity curve
TSPO: Translocator protein
