# Supplementary figures and images for: In vivo TSPO and cannabinoid receptor type 2 availability early in post-stroke neuroinflammation in rats: a positron emission tomography study
Source: J Neuroinflammation. 2017 Mar 29;14:69. doi: 10.1186/s12974-017-0851-4 (PMC5372312; doi:10.1186/s12974-017-0851-4)

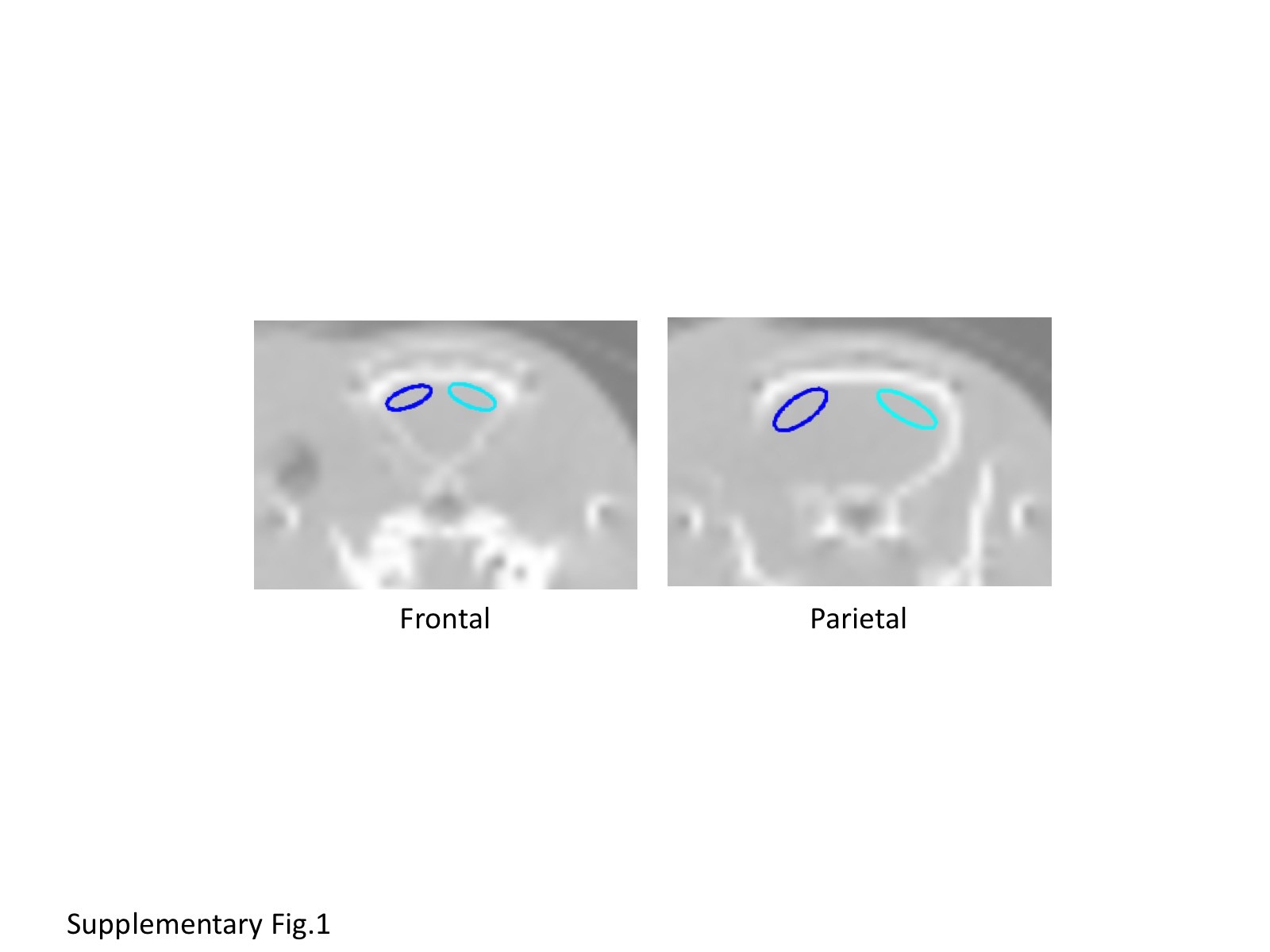

Supplement: Supplementary file 1 — CT images and regions of interest. Frontal, −2 mm from the olfactory-frontal cortex junction (nearly 3 mm from the bregma). Parietal, −6 mm from the junction (nearly −1 mm from the bregma). Blue, lesion side; light blue, contralateral (non-lesion) side. (JPG 68 kb) [file 12974_2017_851_MOESM1_ESM.jpg]

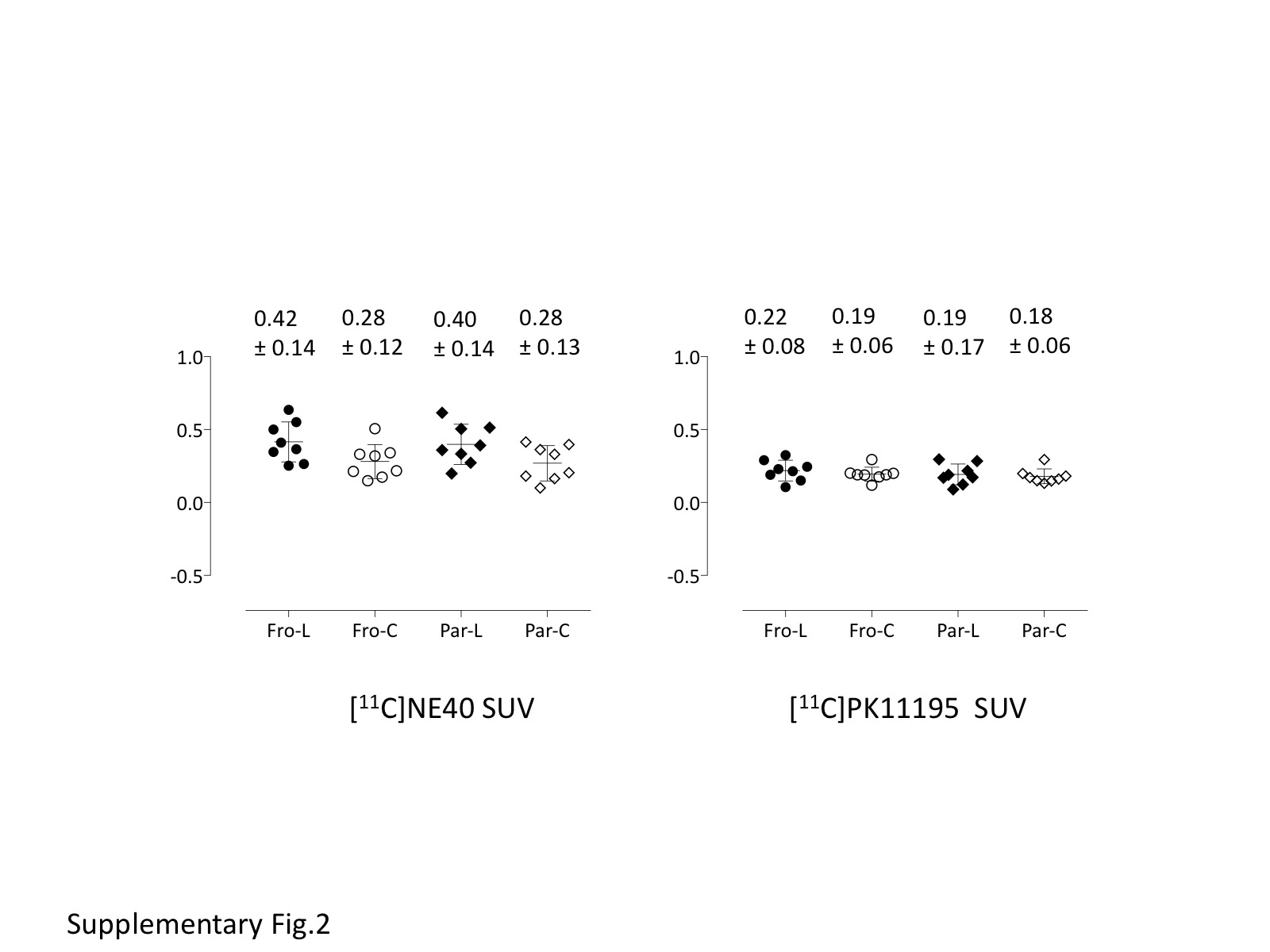

Supplement: Supplementary file 2 — The levels of SUV in each brain region. No significant difference was found in either [11C]NE40 or [11C](R)PK11195 SUV levels. (JPG 100 kb) [file 12974_2017_851_MOESM2_ESM.jpg]

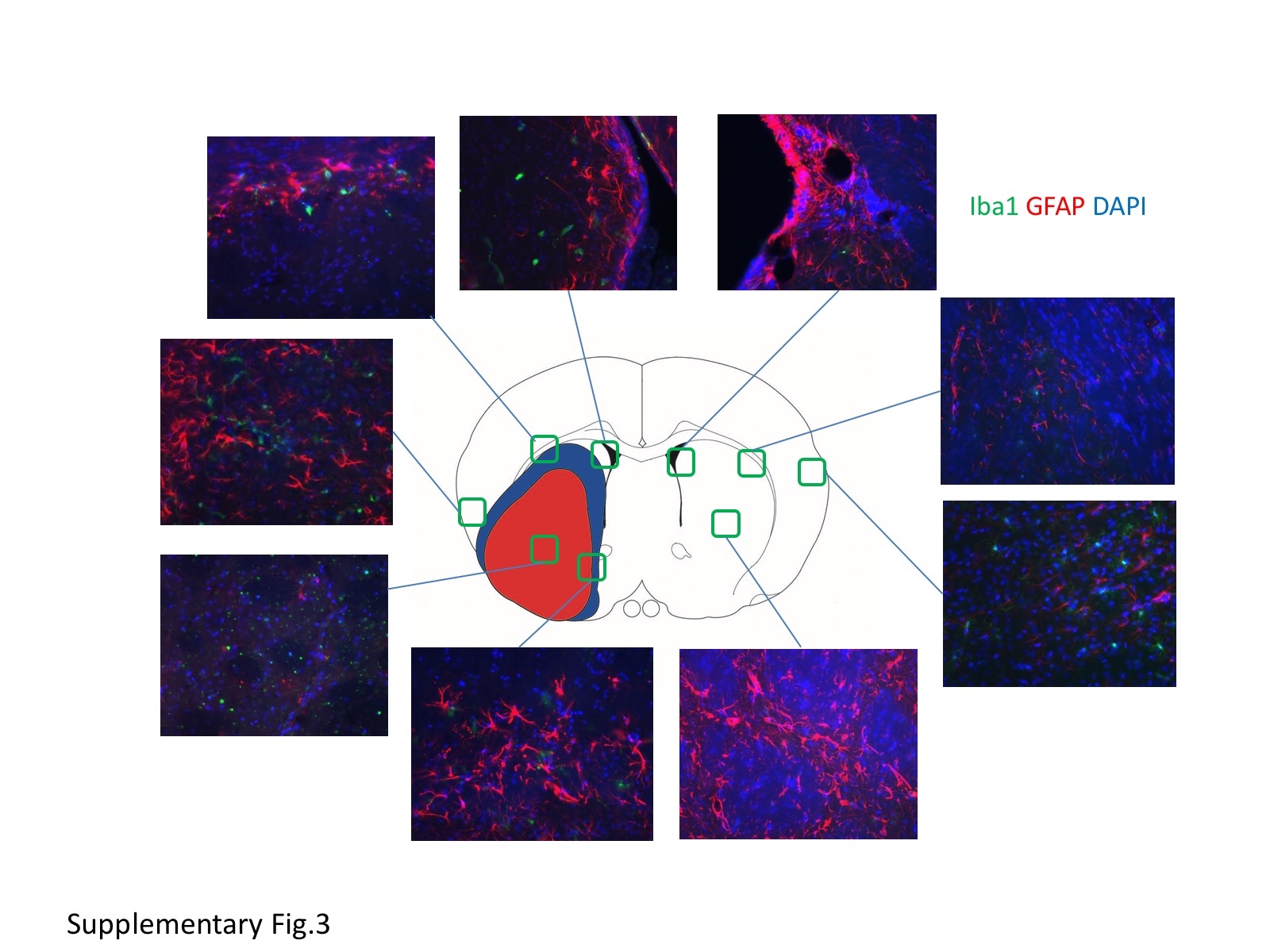

Supplement: Supplementary file 3 — Immunohistochemical results one day after photochemically induced thrombosis (PIT). Iba-1- and GFAP-positive regions are scattered around the infarct core. (JPG 248 kb) [file 12974_2017_851_MOESM3_ESM.jpg]
